# 2-Hy­droxy-*N*′-(4-hy­droxy­benzyl­idene)-3-methyl­benzohydrazide

**DOI:** 10.1107/S1600536811055930

**Published:** 2012-01-07

**Authors:** Xi-Hai Shen, Li-Xue Zhu, Li-Juen Shao, Zhao-Fu Zhu

**Affiliations:** a Department of Chemistry, Hebei Normal University of Science and Technology, Qinhuangdao 066600, People’s Republic of China

## Abstract

The title compound, C_15_H_14_N_2_O_3_, was prepared by condensing 4-hy­droxy­benzaldehyde and 2-hy­droxy-3-methyl­benzo­hydra­zide in methanol. The two benzene rings make a dihedral angle of 19.03 (11)°. An intra­molecular O—H⋯O hydrogen bond is observed. The crystal structure is stabilized by inter­molecular O—H⋯O and N—H⋯O hydrogen bonds and C—H⋯O inter­actions, which lead to the formation of a three-dimensional network.

## Related literature

For the crystal structures of similar hydrazone compounds, see: Fun *et al.* (2011 ▶); Horkaew *et al.* (2011 ▶); Zhi *et al.* (2011 ▶); Huang & Wu (2010 ▶).
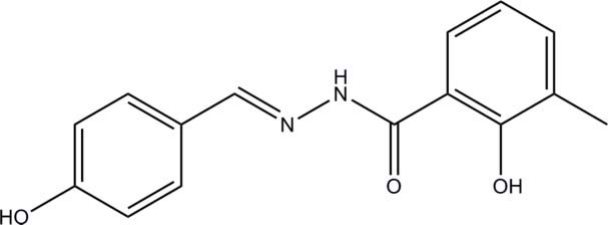


## Experimental

### 

#### Crystal data

C_15_H_14_N_2_O_3_
*M* *_r_* = 270.28
Orthorhombic, 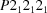

*a* = 7.3872 (17) Å
*b* = 13.012 (2) Å
*c* = 13.592 (2) Å
*V* = 1306.5 (4) Å^3^
*Z* = 4
Mo *K*α radiation
μ = 0.10 mm^−1^
*T* = 298 K
0.20 × 0.20 × 0.17 mm

#### Data collection

Bruker SMART CCD area-detector diffractometer
Absorption correction: multi-scan (*SADABS*; Bruker, 2001 ▶) *T*_min_ = 0.981, *T*_max_ = 0.984
6296 measured reflections
2663 independent reflections
1953 reflections with *I* > 2σ(*I*)
*R*_int_ = 0.032

#### Refinement

*R*[*F* ^2^ > 2σ(*F* ^2^)] = 0.044
*wR*(*F* ^2^) = 0.103
*S* = 0.99
2663 reflections
184 parameters
H-atom parameters constrained
Δρ_max_ = 0.13 e Å^−3^
Δρ_min_ = −0.19 e Å^−3^

### 

Data collection: *SMART* (Bruker, 2007 ▶); cell refinement: *SAINT* (Bruker, 2007 ▶); data reduction: *SAINT*; program(s) used to solve structure: *SHELXS97* (Sheldrick, 2008 ▶); program(s) used to refine structure: *SHELXL97* (Sheldrick, 2008 ▶); molecular graphics: *SHELXTL* (Sheldrick, 2008 ▶); software used to prepare material for publication: *SHELXTL*.

## Supplementary Material

Crystal structure: contains datablock(s) global, I. DOI: 10.1107/S1600536811055930/su2356sup1.cif

Structure factors: contains datablock(s) I. DOI: 10.1107/S1600536811055930/su2356Isup2.hkl

Supplementary material file. DOI: 10.1107/S1600536811055930/su2356Isup3.cml

Additional supplementary materials:  crystallographic information; 3D view; checkCIF report

## Figures and Tables

**Table 1:** Hydrogen-bond geometry (Å, °)

*D*—H⋯*A*	*D*—H	H⋯*A*	*D*⋯*A*	*D*—H⋯*A*
O3—H3⋯O2	0.82	1.86	2.575 (2)	146
O1—H1⋯O2^i^	0.82	2.00	2.809 (2)	167
N2—H2*A*⋯O1^ii^	0.86	2.36	3.067 (2)	139
C3—H3*A*⋯O2^i^	0.93	2.51	3.208 (2)	132

Symmetry codes: (i) 
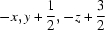
; (ii) 
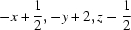
.

**Fig. 1.**
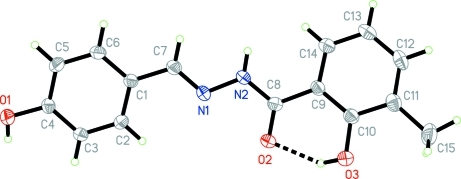
The molecular structure of the title molecule, with atom numbering and displacement ellipsoids drawn at the 30% probability level. The intramolecular O—H···O hydrogen bond is drawn as a dashed line.

**Fig. 2.**
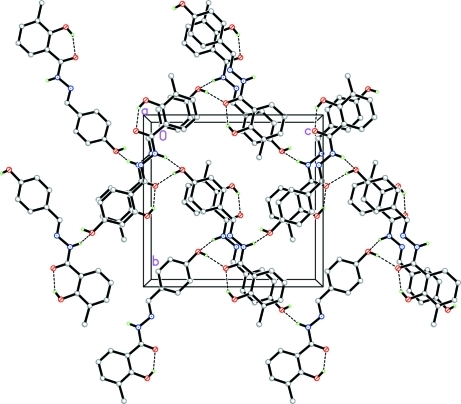
The crystal packing of the title compound, viewed along the a axis. The O-H···O and N-H···O hydrogen bonds are drawn as dashed lines.

## supplementary crystallographic information

### Comment

In the last few years, the crystal structures of a number of hydrazone compounds have been reported (Fun *et al.*, 2011; Horkaew *et al.*, 2011; Zhi *et al.*, 2011; Huang & Wu, 2010). As an extension of work on such compounds, we report herein on the synthesis and crystal structure of the title compound.

In the title molecule, Fig. 1, there is an intramolecular O3—H3···O2 hydrogen bond (Table 1). The benzene rings, (C1—C6) and (C9—C14), make a dihedral angle of 19.03 (11)°. All the geometrical parameters are within normal ranges and are comparable with those in similar compounds, mentioned above.

In the crystal, molecules are linked via O—H···O and N—H···O hydrogen bonds and C-H···O interactions, leading to the formation of a three-dimensional network (Table 1 and Fig. 2).

### Experimental

4-Hydroxybenzaldehyde (122.1 mg, 1.0 mmol) and 2-hydroxy-3-methylbenzohydrazide (166.2 mg, 1.0 mmol) were mixed in methanol (60 ml). The mixture was refluxed for 30 min, then cooled to room temperature, yielding a colourless solution. Colourless crystals were formed when the solution was left to evaporate in air for several days.

### Refinement

All the H atoms were placed in calculated positions and refined as riding atoms: O—H = 0.82 Å, N—H = 0.86 Å, C—H = 0.93 and 0.96 Å for CH and CH_3_ H atoms, respectively, with U_iso_(H) = k × U_eq_(O,N,C), where k = 1.5 for OH and CH_3_ H-atoms and k = 1.2 for all other H-atoms. In the absence of significant anomalous scattering effects the Flack parameter of 2.5 (15) for 1092 Friedel pairs, has no meaning.

### Crystal data

**Table:**

C_15_H_14_N_2_O_3_	*F*(000) = 568
*M**_r_* = 270.28	*D*_x_ = 1.374 Mg m^−^^3^
Orthorhombic, *P*2_1_2_1_2_1_	Mo *K*α radiation, λ = 0.71073 Å
Hall symbol: P 2ac 2ab	Cell parameters from 1375 reflections
*a* = 7.3872 (17) Å	θ = 2.6–24.5°
*b* = 13.012 (2) Å	µ = 0.10 mm^−^^1^
*c* = 13.592 (2) Å	*T* = 298 K
*V* = 1306.5 (4) Å^3^	Block, colourless
*Z* = 4	0.20 × 0.20 × 0.17 mm

### Data collection

**Table:**

Bruker SMART CCD area-detector diffractometer	2663 independent reflections
Radiation source: fine-focus sealed tube	1953 reflections with *I* > 2σ(*I*)
graphite	*R*_int_ = 0.032
ω scans	θ_max_ = 26.5°, θ_min_ = 3.0°
Absorption correction: multi-scan (*SADABS*; Bruker, 2001)	*h* = −9→8
*T*_min_ = 0.981, *T*_max_ = 0.984	*k* = −13→16
6296 measured reflections	*l* = −17→15

### Refinement

**Table:**

Refinement on *F*^2^	Primary atom site location: structure-invariant direct methods
Least-squares matrix: full	Secondary atom site location: difference Fourier map
*R*[*F*^2^ > 2σ(*F*^2^)] = 0.044	Hydrogen site location: inferred from neighbouring sites
*wR*(*F*^2^) = 0.103	H-atom parameters constrained
*S* = 0.99	*w* = 1/[σ^2^(*F*_o_^2^) + (0.0494*P*)^2^] where *P* = (*F*_o_^2^ + 2*F*_c_^2^)/3
2663 reflections	(Δ/σ)_max_ = 0.001
184 parameters	Δρ_max_ = 0.13 e Å^−^^3^
0 restraints	Δρ_min_ = −0.19 e Å^−^^3^

### Special details

**Table:**

Geometry. All e.s.d.'s (except the e.s.d. in the dihedral angle between two l.s. planes) are estimated using the full covariance matrix. The cell e.s.d.'s are taken into account individually in the estimation of e.s.d.'s in distances, angles and torsion angles; correlations between e.s.d.'s in cell parameters are only used when they are defined by crystal symmetry. An approximate (isotropic) treatment of cell e.s.d.'s is used for estimating e.s.d.'s involving l.s. planes.
Refinement. Refinement of *F*^2^ against ALL reflections. The weighted *R*-factor *wR* and goodness of fit *S* are based on *F*^2^, conventional *R*-factors *R* are based on *F*, with *F* set to zero for negative *F*^2^. The threshold expression of *F*^2^ > σ(*F*^2^) is used only for calculating *R*-factors(gt) *etc*. and is not relevant to the choice of reflections for refinement. *R*-factors based on *F*^2^ are statistically about twice as large as those based on *F*, and *R*- factors based on ALL data will be even larger.

### Fractional atomic coordinates and isotropic or equivalent isotropic displacement parameters (Å^2^)

**Table:**

	*x*	*y*	*z*	*U*_iso_*/*U*_eq_	
N1	0.0995 (3)	0.79013 (12)	0.51891 (12)	0.0453 (5)	
N2	0.1159 (3)	0.72214 (12)	0.44083 (12)	0.0455 (5)	
H2A	0.1404	0.7437	0.3825	0.055*	
O1	0.1126 (2)	1.18743 (10)	0.79368 (10)	0.0516 (4)	
H1	0.0544	1.1672	0.8412	0.077*	
O2	0.0499 (2)	0.59172 (10)	0.54350 (10)	0.0523 (5)	
O3	0.1334 (3)	0.40715 (11)	0.49251 (11)	0.0600 (5)	
H3	0.1016	0.4528	0.5303	0.090*	
C1	0.1268 (3)	0.96163 (14)	0.57681 (14)	0.0376 (5)	
C2	0.0866 (3)	0.93585 (16)	0.67358 (15)	0.0430 (6)	
H2	0.0608	0.8679	0.6894	0.052*	
C3	0.0846 (3)	1.00949 (16)	0.74632 (14)	0.0433 (6)	
H3A	0.0602	0.9908	0.8110	0.052*	
C4	0.1188 (3)	1.11133 (14)	0.72333 (15)	0.0395 (5)	
C5	0.1604 (3)	1.13823 (17)	0.62779 (15)	0.0479 (6)	
H5	0.1856	1.2063	0.6121	0.057*	
C6	0.1646 (3)	1.06390 (16)	0.55602 (16)	0.0457 (6)	
H6	0.1935	1.0826	0.4919	0.055*	
C7	0.1324 (3)	0.88408 (15)	0.49972 (15)	0.0424 (5)	
H7	0.1604	0.9034	0.4356	0.051*	
C8	0.0922 (3)	0.62132 (15)	0.45912 (15)	0.0410 (5)	
C9	0.1136 (3)	0.54807 (15)	0.37750 (14)	0.0393 (5)	
C10	0.1344 (3)	0.44378 (16)	0.39901 (15)	0.0437 (5)	
C11	0.1573 (3)	0.37050 (17)	0.32441 (17)	0.0487 (6)	
C12	0.1510 (3)	0.40437 (19)	0.22839 (17)	0.0541 (6)	
H12	0.1634	0.3569	0.1777	0.065*	
C13	0.1266 (3)	0.50748 (19)	0.20530 (17)	0.0537 (6)	
H13	0.1215	0.5281	0.1399	0.064*	
C14	0.1101 (3)	0.57847 (17)	0.27877 (15)	0.0465 (5)	
H14	0.0964	0.6476	0.2630	0.056*	
C15	0.1874 (4)	0.26079 (17)	0.35184 (19)	0.0692 (8)	
H15A	0.2038	0.2206	0.2933	0.104*	
H15B	0.0842	0.2357	0.3875	0.104*	
H15C	0.2934	0.2555	0.3924	0.104*	

### Atomic displacement parameters (Å^2^)

**Table:**

	*U*^11^	*U*^22^	*U*^33^	*U*^12^	*U*^13^	*U*^23^
N1	0.0612 (13)	0.0413 (10)	0.0334 (10)	0.0007 (9)	0.0050 (9)	−0.0060 (7)
N2	0.0655 (13)	0.0413 (10)	0.0297 (9)	0.0004 (9)	0.0056 (10)	−0.0025 (8)
O1	0.0721 (12)	0.0415 (8)	0.0411 (8)	−0.0051 (8)	0.0055 (8)	−0.0065 (7)
O2	0.0801 (12)	0.0455 (9)	0.0312 (8)	−0.0016 (8)	0.0081 (8)	0.0011 (7)
O3	0.0948 (13)	0.0435 (9)	0.0416 (9)	0.0010 (10)	0.0016 (10)	0.0033 (7)
C1	0.0427 (12)	0.0367 (11)	0.0335 (11)	0.0039 (10)	−0.0002 (10)	0.0008 (9)
C2	0.0518 (14)	0.0346 (11)	0.0427 (12)	−0.0004 (10)	0.0029 (11)	0.0039 (10)
C3	0.0576 (16)	0.0410 (12)	0.0313 (11)	0.0001 (10)	0.0038 (11)	0.0010 (9)
C4	0.0436 (13)	0.0366 (11)	0.0383 (12)	0.0026 (10)	0.0018 (11)	−0.0053 (9)
C5	0.0598 (15)	0.0362 (11)	0.0475 (14)	−0.0068 (10)	0.0091 (12)	0.0031 (10)
C6	0.0579 (15)	0.0453 (13)	0.0339 (11)	0.0019 (11)	0.0046 (12)	0.0053 (10)
C7	0.0469 (13)	0.0434 (13)	0.0368 (12)	0.0037 (11)	0.0051 (11)	−0.0005 (10)
C8	0.0458 (13)	0.0425 (12)	0.0347 (12)	−0.0010 (10)	−0.0004 (11)	0.0019 (9)
C9	0.0429 (12)	0.0411 (11)	0.0340 (11)	−0.0024 (10)	−0.0009 (11)	−0.0042 (9)
C10	0.0491 (14)	0.0453 (12)	0.0365 (12)	−0.0060 (11)	−0.0005 (11)	−0.0023 (10)
C11	0.0458 (14)	0.0508 (14)	0.0495 (14)	−0.0061 (11)	0.0031 (11)	−0.0102 (11)
C12	0.0519 (15)	0.0624 (16)	0.0481 (14)	−0.0065 (12)	0.0039 (12)	−0.0237 (12)
C13	0.0602 (16)	0.0687 (16)	0.0322 (12)	−0.0023 (14)	0.0010 (12)	−0.0033 (11)
C14	0.0520 (14)	0.0515 (13)	0.0360 (12)	0.0003 (11)	−0.0003 (11)	0.0011 (10)
C15	0.083 (2)	0.0445 (14)	0.0800 (19)	−0.0051 (13)	0.0022 (16)	−0.0157 (13)

### Geometric parameters (Å, °)

**Table:**

N1—C7	1.273 (2)	C5—H5	0.9300
N1—N2	1.387 (2)	C6—H6	0.9300
N2—C8	1.347 (2)	C7—H7	0.9300
N2—H2A	0.8600	C8—C9	1.471 (3)
O1—C4	1.377 (2)	C9—C10	1.397 (3)
O1—H1	0.8200	C9—C14	1.399 (3)
O2—C8	1.249 (2)	C10—C11	1.402 (3)
O3—C10	1.357 (2)	C11—C12	1.378 (3)
O3—H3	0.8200	C11—C15	1.492 (3)
C1—C6	1.389 (3)	C12—C13	1.390 (3)
C1—C2	1.389 (3)	C12—H12	0.9300
C1—C7	1.455 (3)	C13—C14	1.366 (3)
C2—C3	1.377 (3)	C13—H13	0.9300
C2—H2	0.9300	C14—H14	0.9300
C3—C4	1.385 (3)	C15—H15A	0.9600
C3—H3A	0.9300	C15—H15B	0.9600
C4—C5	1.380 (3)	C15—H15C	0.9600
C5—C6	1.374 (3)		
			
C7—N1—N2	116.04 (18)	O2—C8—N2	120.15 (18)
C8—N2—N1	117.96 (17)	O2—C8—C9	121.28 (18)
C8—N2—H2A	121.0	N2—C8—C9	118.56 (18)
N1—N2—H2A	121.0	C10—C9—C14	118.51 (19)
C4—O1—H1	109.5	C10—C9—C8	118.92 (18)
C10—O3—H3	109.5	C14—C9—C8	122.55 (18)
C6—C1—C2	117.84 (18)	O3—C10—C9	122.45 (19)
C6—C1—C7	120.81 (18)	O3—C10—C11	116.04 (19)
C2—C1—C7	121.34 (18)	C9—C10—C11	121.5 (2)
C3—C2—C1	120.93 (18)	C12—C11—C10	117.6 (2)
C3—C2—H2	119.5	C12—C11—C15	123.2 (2)
C1—C2—H2	119.5	C10—C11—C15	119.2 (2)
C2—C3—C4	120.13 (19)	C11—C12—C13	121.8 (2)
C2—C3—H3A	119.9	C11—C12—H12	119.1
C4—C3—H3A	119.9	C13—C12—H12	119.1
O1—C4—C5	118.58 (18)	C14—C13—C12	120.0 (2)
O1—C4—C3	121.69 (18)	C14—C13—H13	120.0
C5—C4—C3	119.73 (18)	C12—C13—H13	120.0
C6—C5—C4	119.64 (19)	C13—C14—C9	120.6 (2)
C6—C5—H5	120.2	C13—C14—H14	119.7
C4—C5—H5	120.2	C9—C14—H14	119.7
C5—C6—C1	121.70 (19)	C11—C15—H15A	109.5
C5—C6—H6	119.1	C11—C15—H15B	109.5
C1—C6—H6	119.1	H15A—C15—H15B	109.5
N1—C7—C1	120.85 (19)	C11—C15—H15C	109.5
N1—C7—H7	119.6	H15A—C15—H15C	109.5
C1—C7—H7	119.6	H15B—C15—H15C	109.5

### Hydrogen-bond geometry (Å, °)

**Table:**

*D*—H···*A*	*D*—H	H···*A*	*D*···*A*	*D*—H···*A*
O3—H3···O2	0.82	1.86	2.575 (2)	146
O1—H1···O2^i^	0.82	2.00	2.809 (2)	167
N2—H2A···O1^ii^	0.86	2.36	3.067 (2)	139
C3—H3A···O2^i^	0.93	2.51	3.208 (2)	132

Symmetry codes: (i) −*x*, *y*+1/2, −*z*+3/2; (ii) −*x*+1/2, −*y*+2, *z*−1/2.
